# Prognostic psychosocial factors for disabling low back pain in Japanese hospital workers

**DOI:** 10.1371/journal.pone.0177908

**Published:** 2017-05-22

**Authors:** Takahiko Yoshimoto, Hiroyuki Oka, Junji Katsuhira, Tomoko Fujii, Katsuhiko Masuda, Sakae Tanaka, Ko Matsudaira

**Affiliations:** 1 Department of Rehabilitation, Kameda Medical Center, Chiba, Japan; 2 Department of Medical Research and Management for Musculoskeletal Pain, 22nd Century Medical & Research Center, the University of Tokyo, Tokyo, Japan; 3 Department of Prosthetics & Orthotics and Assistive Technology, Faculty of Medical Technology, Niigata University of Health and Welfare, Niigata, Japan; 4 Incorporated Medical Institution Yoseikai, Tokyo, Japan; 5 Department of Orthopaedic Surgery, University of Tokyo, Tokyo, Japan; Universita Cattolica del Sacro Cuore Sede di Roma, ITALY

## Abstract

**Background:**

Although the occupational health field has identified psychosocial factors as risk factors for low back pain that causes disability, the association between disabling low back pain and psychosocial factors has not been examined adequately in Japanese hospital workers. Therefore, this study examined the association between low back pain, which interfered with work, and psychosocial factors in Japanese hospital workers.

**Method:**

This cross-sectional study was conducted at a hospital in Japan. In total, 280 hospital workers were recruited from various occupational settings. Of these, 203 completed a self-administered questionnaire that included items concerning individual characteristics, severity of low back pain, fear-avoidance beliefs (Fear-Avoidance Beliefs Questionnaire), somatic symptoms (Somatic Symptom Scale-8), psychological distress (K6), workaholism, and work-related psychosocial factors (response rate: 72.5%). Logistic regression was used to explore risk factors associated with disabling low back pain.

**Results:**

Of the 203 participants who completed questionnaires, 36 (17.7%) reported low back pain that interfered with their work. Multivariate analyses with individual factors and occupations adjusted for showed statistically significant associations between disabling low back pain and fear-avoidance beliefs (adjusted odds ratio [OR]: 2.619, 95% confidence interval [CI]: 1.003–6.538], somatic symptoms (OR: 4.034, 95% CI: 1.819–9.337), and interpersonal stress at work (OR: 2.619, 95% CI: 1.067–6.224).

**Conclusions:**

Psychosocial factors, such as fear-avoidance beliefs, somatic symptoms, and interpersonal relationships at work, were important risk factors in low back pain that interfered with work in Japanese hospital workers. With respect to occupational health, consideration of psychosocial factors is required to reduce disability related to low back pain.

## Introduction

Low back pain (LBP) is an extremely common global health problem [1] and one of the main causes of disability in working populations [2]. According to the Global Burden of Disease Study conducted in 2013, which was an international collaborative effort led by the Institute for Health Metrics and Evaluation to quantify the absolute and relative burden of ill health and estimate prevalences and years lived with disability for 301 diseases and injuries, LBP was the leading cause of disability [3]. LBP is also considered a socioeconomic problem in the occupational health field [4–7]. A previous study examining the economic impact of various health conditions on work performance in Japanese workers indicated that LBP was one of the primary health conditions leading to work loss [8]. In addition, a large-scale survey examining LBP prevalence and associated factors in the Japanese adult population showed that one in four workers had been absent from work or other activities because of LBP [9]. The number of workers who were absent from social activities for at least 4 consecutive days has increased annually in the public health and hygiene fields [10]; therefore, the establishment of effective methods for the prevention of LBP in the workplace is urgently required.

In contrast, the understanding and interpretation of LBP is commonly based on the biopsychosocial model, and the importance of both psychosocial and biomedical factors has been emphasized in the development and persistence of LBP [11–13] In addition, the occupational health field has shown that psychosocial aspects of work play an important role in LBP chronicity and LBP-related disability [14]. Of the psychosocial factors related to LBP, fear-avoidance beliefs (FABs) in the management of LBP have received considerable global attention [15–17]. In addition, work-related psychosocial factors [18–23], such as job satisfaction, worksite support, interpersonal stress, workaholism, and a tendency toward somatization [24], have been associated with the development of LBP. However, few studies have examined the relationship between LBP and psychosocial factors in Japanese public health service workers systematically. The aim of this cross-sectional study was to explore LBP in medical and nonmedical hospital workers and perform a systematic examination of the association between psychosocial factors and LBP with disability.

## Materials and methods

### Study population

Cross-sectional data collected from the baseline survey of the Yoseikai Study, an occupational cohort study conducted in Japan, were used in the current study.

The research ethics committee for the Graduate School of Medicine and Faculty of Medicine at the University of Tokyo (No. 1264) and the Incorporated Medical Institution Yoseikai reviewed and approved the study’s aim and procedure. Written informed consent was obtained from all participants prior to the initiation of the study.

In March 2015, all of the employees at a Japanese hospital (N = 280) were recruited via an invitation letter from the authors. The survey was conducted during May 2015. During the survey period, occupational health staff at the hospital distributed a nonanonymous, self-administered questionnaire to each employee. Once the employees had completed the questionnaires, they placed them in sealed envelopes, which occupational health staff collected and forwarded to the authors. All employees were assured that their participation was voluntary, and supervisors and occupational health staff were not authorized to open the sealed envelopes. In total, 203 employees completed the self-administered questionnaire.

### Study measures

The questionnaire included questions regarding the following: individual characteristics including sex, age, body mass index (BMI), and occupation type; LBP severity; and individual and work-related psychosocial factors. LBP severity was evaluated by the respondents, who were asked to indicate the severity of their LBP according to four grades (0 = no LBP, 1 = LBP that did not interfere with work, 2 = LBP that interfered with work, and 3 = LBP that interfered with work and required sick leave). The grades were determined with reference to Von Korff’s grading method [25]. LBP was defined as pain in the lower back lasting for more than 1 day and experienced during the preceding 4 weeks, according to the standard definition of LBP proposed by Dionne et al. [26]. Pain associated with menstruation or pregnancy or experienced during a feverish illness was excluded. A diagram showing the lower back area (between the inferior costal margin and gluteal folds [11]) was included in the questionnaire. LBP with disability was defined as LBP that interfered with work, regardless of work attendance (Grade 2 or 3), because presenteeism, or working while unwell, can lead to productivity loss and poor health.

The questions concerning current occupation type pertained to job satisfaction, job demand, job control, interpersonal stress at work, and social support. Work-related stress was assessed using the Brief Job Stress Questionnaire [27,28], which was developed by a research working group established by the Japan Labour, Health and Welfare Organization. The scale contains 57 items measuring psychosocial work environments, stress reactions, and buffering factors, with responses provided using a four-point Likert scale ranging from 1 to 4, with reverse scoring applied to some items. Total scores range from 57 to 228, and higher scores indicate greater work-related stress. The five original responses were reclassified as “not stressed,” which included low, slightly low, and moderate stress, and “stressed,” which included slightly high and high stress [20,23].

We evaluated mental health problems using the Kessler Screening Scale for Psychological Distress (K6), which was developed in 2002 as a short-form version of the K10 [29]. The scale measures psychological distress experienced during the preceding 30 days, using six items, with responses provided using a five–point scale ranging from 0 (all of the time) to 4 (none of the time) or ranging from 1 (all of the time) to 5 (none of the time). The Japanese version of the scale was developed by Furukawa et al. in 2008 and demonstrated reliability and validity [30]. Respondents were classified into three groups according to their total scores (≥10 = high, 5–9 = moderate, and ≤4 = low).

Somatic symptom burden was measured using the Somatic Symptoms Scale-8 (SSS-8), which is an abbreviated eight-item version of the Patient Health Questionnaire-15. We used the linguistically validated Japanese version of the SSS-8, which was developed in our previous study [31]. The scale measures the extent to which respondents have been bothered by somatic symptoms during the preceding 7 days, with responses provided using a five-point Likert scale ranging from 0 (not at all) to 4 (very much). Total scores range from 0 to 32 and represent somatic symptom severity, with ≥16 points indicating very severe symptoms [32].

Participants’ beliefs and fears were measured using the Fear-Avoidance Beliefs Questionnaire (FABQ), which was developed by Waddell et al. [33] and consists of 16 self-reported items. We used the Japanese version of the FABQ, which was developed and validated recently by Matsudaira et al. [34]. The study used the FABQ’s four-item physical activity subscale (FABQ-PA), which measures respondents’ beliefs about the effects of physical activity on their LBP. Responses are provided using a seven-point Likert scale ranging from 0 (completely disagree) to 6 (completely agree). Total scores range from 0 to 24, and higher scores represent higher FAB levels. Participants’ scores were classified into two categories (≤14 = low, ≥15 = high) [35].

Workaholism, which has been associated with psychological health, was measured using the Dutch Workaholism Scale [36], which consists of two subscales: working excessively and working compulsively. Each subscale consists of five items, with responses provided using a four-point Likert scale ranging from 1 (totally disagree) to 4 (totally agree). Respondents were classified into three groups according to their total scores (high, moderate, and low) [21].

### Statistical analysis

We performed logistic regression analysis, as our dependent variable “presence or absence of chronic pain” was dichotomous. One set of guidelines suggested that accurate estimation of discriminant function parameters requires a sample size of at least 20 for each independent variable in logistic regression [37]. In addition, the prevalence rates for chronic pain reportedly range from 10% to 55% [38]. Therefore, we calculated an overall sample size of 200 to ensure that there were 20 participants, even with a minimum prevalence rate of 10% for chronic pain.

Demographic and clinical characteristics were compared using Student's t test for continuous variables and chi-square tests for categorical variables. Factors associated with LBP were assessed using multivariable logistic regression analysis. Risk factors included job satisfaction; job demand; job stress; job control; social support from a supervisor/manager, colleagues, or family; K6 score; SSS-8 score; workaholism score; and FABQ-PA score. Because of the relatively low number of participants with back pain in the study, propensity score adjustment was used for each of the risk factors in multivariate modeling.

Propensity score adjustment preserved statistical power by reducing covariates into a single variable. For example, when the adjusted effect of LBP was evaluated, a propensity score was created using binary logistic regression to predict the probability of LBP as a function of the important factors (sex, age, BMI, occupation type [medical or nonmedical]) included in the study. Data analysis was performed using SAS software (version 9.4, SAS Institute Inc., Cary, NC).

## Results

Participants’ characteristics are shown in Table 1. Their mean age was 39.8 (SD = 12.2) years, and 70% of the participants were women. Most (63.1%) participants’ occupation types were classified as medical, and their mean BMI score was 22.6 (SD = 4.1).

**Table 1:** Participant characteristics (N = 203).

		n (%)
Sex	Male	61 (30.0)
	Female	142 (70.0)
Occupation type	Medical	128 (63.1)
	Nonmedical	75 (36.9)
FABQ-PA score	Low	172 (85.6)
	High	29 (14.4)
Job satisfaction	Dissatisfied	51 (26.2)
	Satisfied	144 (73.8)
Job demand	Not stressed	126 (62.7)
	Stressed	75 (37.3)
Interpersonal stress at work	Not stressed	163 (81.5)
	Stressed	37 (18.5)
Job control	Control	147 (72.8)
	No control	55 (27.2)
Support from supervisors	Supported	114 (57.6)
	Unsupported	84 (42.4)
Support from coworkers	Supported	151 (75.9)
	Unsupported	48 (24.1)
Support from family and friends	Supported	58 (29.1)
	Unsupported	141 (70.9)
K6 score	Low	103 (50.7)
	Moderate	54 (26.6)
	High	46 (22.7)
SSS-8 score	Other	128 (64.0)
	Very high	72 (36.0)
Workaholism score	Low	63 (31.2)
	Moderate	73 (36.1)
	High	66 (32.7)

BMI: body mass index; FABQ-PA: Fear-Avoidance Beliefs Questionnaire-Physical Activity; LBP: low back pain; SD: standard deviation; SSS8: Somatic Symptom Scale-8

Of the 203 participants who responded to the questionnaires, 36 (17.7%) reported LBP that interfered with their work. The results of the comparison of characteristics between participants with and without LBP are shown in Table 2. Participants without LBP (mean age = 44.3, SD = 10.4 years) were significantly older relative to those with LBP (mean age = 38.8, SD = 12.3 years, p = .013). BMI did not differ significantly between participants with (M = 22.9, SD = 4.4) and without LBP (M = 22.5, SD = 4.0; p = .590). FABQ-PA scores (p = .037), SSS-8 scores (p < .001), and interpersonal stress at work (p = .022) in participants with LBP that did not interfere with work were significantly higher relative to those observed in those with LBP that interfered with work.

**Table 2:** Comparison of characteristics between participants with and without LBP.

Factors		With LBP (n)	Without LBP (n)	p value
n		36	167	
Sex	Male	15	46	.094
	Female	21	121	
Occupation type	Medical	26	102	.209
	Nonmedical	10	65	
FABQ-PA score	Low	26	146	.037
	High	9	20	
Job satisfaction	Dissatisfied	10	41	.634
	Satisfied	24	120	
Job demand	Not stressed	19	107	.258
	Stressed	16	59	
Interpersonal stress at work	Not stressed	23	140	.022
	Stressed	11	26	
Job control	Control	26	121	.825
	No control	9	46	
Support from supervisors	Supported	21	93	.440
	Unsupported	12	72	
Support from coworkers	Supported	28	123	.333
	Unsupported	6	42	
Support from family and friends	Supported	10	48	.970
	Unsupported	24	117	
K6 score	Low	18	85	.794
	Moderate	11	43	
	High	7	39	
SSS-8 score	Other	13	115	.0003
	Very high	22	50	
Workaholism score	Low	10	53	.678
	Moderate	12	61	
	High	14	52	

BMI: body mass index; FABQ: Fear-Avoidance Beliefs Questionnaire-Physical Activity; LBP: low back pain; SD: standard deviation; SSS8: Somatic Symptom Scale-8

These three variables were extracted from the multiple logistic regression model as significant independent factors, with age, sex, BMI, and occupation type controlled for (Table 3). FABQ-PA scores (adjusted odds ratio [AOR] = 2.619, 95% confidence interval [CI]: 1.003–6.538), SSS-8 scores (AOR = 4.034, 95% CI: 1.819–9.337), and interpersonal stress at work (AOR = 2.619, 95% CI: 1.067–6.224) were significantly associated with LBP that interfered with work.

**Table 3:** Multiple logistic regression analysis of associations between LBP that interfered with work and independent variables.

Factors		Adjusted OR	95% CI	p value
Job satisfaction		1.027	0.436–2.591	.952
Job demand		1.593	0.720–3.503	.248
Interpersonal stress at work		2.619	1.067–6.224	.036
Job control		0.888	0.3549–2.062	.788
Support from supervisors		0.774	0.331–1.743	.539
Support from coworkers		0.645	0.223–1.625	.366
Support from family and friends		1.002	0.439–2.421	.995
K6 score	Moderate vs. High	0.599	0.184–1.821	.368
	Low vs. High	0.881	0.303–2.342	.805
	Low vs. Moderate	1.471	0.579–3.637	.408
SSS-8 score		4.034	1.819–9.337	< .001
Workaholism score	Low vs. Moderate	1.133	0.416–3.121	.805
	Low vs. High	1.453	0.576–3.783	.429
	Moderate vs. High	1.282	0.505–3.324	.600
FABQ-PA score		2.619	1.003–6.538	.049

Adjusted for age, sex, body mass index, and occupation type. CI: confidence interval; FABQ-PA: Fear-Avoidance Beliefs Questionnaire-Physical Activity; LBP, low back pain; OR: odds ratio; SSS8: Somatic Symptom Scale-8

## Discussion

This cross-sectional study examined the association between psychosocial factors and LBP that interfered with Japanese medical and nonmedical workers’ ability to work at a hospital. In the multiple logistic regression analysis, in which age, sex, BMI, and occupation type were controlled for, fear-avoidance beliefs, the tendency toward somatization, and interpersonal stress at work were significantly associated with LBP that interfered with work. This was the first study to demonstrate an association between fear-avoidance behavior and LBP with disability in Japanese medical and nonmedical hospital workers.

The prevalence rates for LBP in a previous study [38] that compared chronic pain prevalence rates between various countries were 13%, 6%, and 1.48% for Japan, Thailand, and Myanmar, respectively. These results showed that, in the Asian region, the prevalence of LBP was particularly high in Japan, which is an advanced country. This could reflect differences in cultural backgrounds, which included psychosocial factors. In addition, Sakakibara’s study [38] defined LBP as chronic pain, while the current study defined it as LBP with disability. However, considering that LBP is the most common type of chronic pain, the prevalence rate for LBP in workers with medical occupations, including nursing, in the current study (17.7%) was similar to that observed in Sakakibara’s study [38], and this is a reasonable result.

The current study examined the association between psychosocial factors and LBP that interfered with work, regardless of sick leave. The cost of work loss resulting from a combination of absenteeism and presenteeism due to back disorders was higher relative to that observed for various other health conditions in Japan [8]. In addition, the estimated cost of work loss resulting from presenteeism due to back pain was higher relative to that resulting from absenteeism due to back pain. Our previous international epidemiological study [24] showed that, relative to British workers, Japanese workers were less likely to take sick leave because of musculoskeletal disorders, particularly LBP. Therefore, in our assessment of LBP in Japanese workers, we defined LBP with disability as LBP that interfered with work, regardless of whether sick leave was taken.

### Relationship between FAB and LBP with disability

The fear-avoidance model has been proposed as a representative type of thought process involving the chronicity of LBP. FABs in LBP are negative beliefs and anxiety regarding LBP, which lead to a catastrophizing response in which the worst possible outcome is imagined, causing fear and avoidance of activity and resulting in functional restriction. Of the psychological factors examined as prognostic factors for the development of chronic LBP, FABs have been shown to constitute an important factor and exerted a strong effect on employment conditions and disability prognoses [39]. The introduction of a psychosocial flag system to manage musculoskeletal problems, including LBP, in healthcare and the workplace has been suggested in Western countries. Within this concept, FABs are represented by a yellow flag, and some researchers have recommended that clinical practitioners should judge the involvement of FABs during the early stages of pain and manage them in cooperation with the patient’s workplace [40]. Some studies have shown that FABs led to a tendency toward development of chronic LBP with disability [41–43]. In the present study, the results indicated that FABs were related to LBP in Japanese public health service workers. As a significant independent factor in multiple logistic analysis with certain variables controlled for, FABs were an important factor and should be considered in the management of LBP. In addition, early intervention is important in increasing awareness of FABs in patients with disabling LBP in Japan.

### Relationship between somatic symptoms and LBP with disability

The tendency toward somatization is defined as a predisposition to excessive worry regarding common somatic symptoms, such as headaches, dizziness, and stomach or bowel problems, which could be triggered by mental distress. This tendency has been shown to affect various health conditions (via related behaviors), musculoskeletal pain (particularly multisite pain) [44], and absence from work [45]. The relationship between pain and the tendency toward somatization has been observed in both longitudinal and cross-sectional studies, indicating that this tendency is a predictor, rather than a consequence, of other aspects of health [46].

The tendency toward somatization has been associated with [15,46–48] and identified as a major risk factor for LBP[24]. The association between chronic LBP with disability and a combination of psychosocial factors could be explained by dysfunction in the mesolimbic dopamine system, which controls both pain and pleasure [49,50] When a person experiences painful stimuli, the mesolimbic dopamine system is activated to inhibit pain. However, exposure to chronic, rather than acute, stress, such as anxiety or distress, has recently been suggested to result in hyperalgesia because of the inhibition of mesolimbic dopamine mechanisms. For example, hyperalgesia resulting from chronic stress because of discontentment with life and work could lead to the development of chronic LBP with disability [51]. The results of the current study indicated that the tendency toward somatization was associated with LBP that interfered with work. This could indicate LBP should be managed as brain dysfunction as well as musculoskeletal disease.

### Relationship between interpersonal stress at work and LBP with disability

The relationship between interpersonal stress at work and occupational LBP has attracted attention for some time [18]. In particular, stressful, monotonous work was identified as a predictive factor for new-onset LBP in a cohort study [52]. In addition, a recent 2-year prospective epidemiological study involving 5,310 workers in Japan suggested that work-related stress affected the onset and persistence of LBP with disability [20]. The way in which psychological factors cause LBP remained unclear in the current study; however, two biomechanical (ergonomic) studies showed that psychological stress increased low back compression force during lifting tasks [53,54], which could indicate that stress is associated with increased risk of LBP development. Our results suggested that interpersonal stress at work is an important factor in understanding occupational LBP.

The results showed that fear-avoidance beliefs regarding LBP, which has been recognized worldwide as an important risk factor; the tendency toward somatization, which is a type of stress response; and interpersonal stress at work were associated with LBP with disability in medical workers. According to the results, pain education based on the biopsychosocial model could be inadequate in Japan, even for medical personnel. A recent systematic review examining LBP prevention indicated that exercise combined with education was likely to reduce the risk of LBP [55]. Ergonomic factors and the psychosocial factors examined in the current study should be considered in education regarding LBP prevention.

The present study was subject to some limitations. First, it was conducted at a single hospital; therefore, the generalization of the results is limited. Second, the number of participants with LBP that interfered with their work was low; however, we dealt with this problem statistically using propensity score adjustment. Third, the study was cross-sectional in design; therefore, causation could not be inferred. We plan to conduct an additional cohort study to examine causal associations. Fourth, various chronic pain conditions interfere with work, but the current study considered only LBP. Future research should examine the effects of chronic pain in various parts of the body and compare them to those observed for LBP. Fifth, the results showed that LBP was affected by individuals’ personal relationships; however, the study did not consider factors examined in previous studies (e.g., family environment, nursing, and genetic predisposition).

In conclusion, the results of the present study suggested that psychosocial factors, such as FABs, the tendency toward somatization, and interpersonal stress at work were associated with LBP that interfered with work. Future preventive strategies for reducing LBP in the workplace should include not only biomechanical factors, which are already well understood, but also the management of psychosocial factors.

## Supporting information

S1 File Supporting information. Dataset of this study.
(XLSX)
